# Tumor promoting effect of PDLIM2 downregulation involves mitochondrial ROS, oncometabolite accumulations and HIF-1α activation

**DOI:** 10.1186/s13046-024-03094-9

**Published:** 2024-06-17

**Authors:** Jing-Xing Yang, Yu-Chen Chuang, Jen-Chih Tseng, Yi-Ling Liu, Chao-Yang Lai, Alan Yueh-Luen Lee, Chi-Ying F. Huang, Yi-Ren Hong, Tsung-Hsien Chuang

**Affiliations:** 1 Immunology Research Center, National Health Research Institutes, Zhunan, Miaoli 35053 Taiwan; 2 Department of Medical Laboratory Science and Biotechnology, Asia University, Taichung, 41354 Taiwan; 3 National Institute of Cancer Research, National Health Research Institutes, Zhunan, Miaoli 35053 Taiwan; 4 Institute of Biopharmaceutical Sciences, College of Pharmaceutical Sciences, National Yang Ming Chiao Tung University, Taipei, 11221 Taiwan; 5 Graduate Institute of Medicine, College of Medicine, Kaohsiung Medical University, Kaohsiung, 80708 Taiwan; 6 Department of Life Sciences, National Central University, Zhongli District, Taoyuan City, 32001 Taiwan

**Keywords:** HIF-1α, HIF-1α inhibitor, Mitochondria, PDLIM2, Succinate, Succinate dehydrogenase

## Abstract

**Background:**

Cancer is characterized by dysregulated cellular metabolism. Thus, understanding the mechanisms underlying these metabolic alterations is important for developing targeted therapies. In this study, we investigated the pro-tumoral effect of PDZ and LIM domain 2 (PDLIM2) downregulation in lung cancer growth and its association with the accumulation of mitochondrial ROS, oncometabolites and the activation of hypoxia-inducible factor-1 (HIF-1) α in the process.

**Methods:**

Databases and human cancer tissue samples were analyzed to investigate the roles of PDLIM2 and HIF-1α in cancer growth. DNA microarray and gene ontology enrichment analyses were performed to determine the cellular functions of PDLIM2. Seahorse assay, flow cytometric analysis, and confocal microscopic analysis were employed to study mitochondrial functions. Oncometabolites were analyzed using liquid chromatography–mass spectrometry (LC–MS). A Lewis lung carcinoma (LLC) mouse model was established to assess the in vivo function of PDLIM2 and HIF-1α.

**Results:**

The expression of PDLIM2 was downregulated in lung cancer, and this downregulation correlated with poor prognosis in patients. PDLIM2 highly regulated genes associated with mitochondrial functions. Mechanistically, PDLIM2 downregulation resulted in NF-κB activation, impaired expression of tricarboxylic acid (TCA) cycle genes particularly the succinate dehydrogenase (SDH) genes, and mitochondrial dysfunction. This disturbance contributed to the accumulation of succinate and other oncometabolites, as well as the buildup of mitochondrial reactive oxygen species (mtROS), leading to the activation of hypoxia-inducible factor 1α (HIF-1α). Furthermore, the expression of HIF-1α was increased in all stages of lung cancer. The expression of PDLIM2 and HIF-1α was reversely correlated in lung cancer patients. In the animal study, the orally administered HIF-1α inhibitor, PX-478, significantly reduces PDLIM2 knockdown-promoted tumor growth.

**Conclusion:**

These findings shed light on the complex action of PDLIM2 on mitochondria and HIF-1α activities in lung cancer, emphasizing the role of HIF-1α in the tumor-promoting effect of PDLIM2 downregulation. Additionally, they provide new insights into a strategy for precise targeted treatment by suggesting that HIF-1α inhibitors may serve as therapy for lung cancer patients with PDLIM2 downregulation.

**Supplementary Information:**

The online version contains supplementary material available at 10.1186/s13046-024-03094-9.

## Introduction

Deregulation of cellular metabolism is a hallmark of cancer. Cancer cells exhibit significant alterations in their metabolic pathways compared to normal cells, enabling them to efficiently adapt to metabolic milieu for rapid proliferation and survival [1–3]. Mitochondria play a crucial role in cellular metabolism. The tricarboxylic acid (TCA) cycle, also known as the Krebs cycle or citric acid cycle, is a central metabolic pathway within the mitochondria. It consists of a series of sequential biochemical reactions within the mitochondrial membrane that lead to the conversion of oxidized forms of nicotinamide adenine dinucleotide (NAD^+^) and flavin adenine dinucleotide (FAD) into their reduced NADH and FADH2 forms. These reduced molecules couple the TCA cycle with the respiratory chain (or electron transport chain) leading to the production of water and adenosine triphosphate (ATP). The TCA cycle also generates metabolites, including citrate, α-ketoglutarate (α-KG), succinyl-CoA, succinate, fumarate, malate, and oxaloacetate. These metabolites serve as precursors for the synthesis of sugars, lipids, and amino acids, which are essential building blocks for cells [4–7]. Under certain conditions, such as hypoxia or impaired respiratory chain function, mitochondrial ROS production can increase [8, 9].

Oncometabolites have been demonstrated to play a role in cancer growth and progression. These metabolites are intermediates that accumulate at abnormal levels and can have profound effects on various cellular processes [10–12]. A prominent example is the accumulation of succinate resulting from mutations in genes encoding the enzyme succinate dehydrogenase (SDH) in the TCA cycle. Mutations in these genes have also been shown to increase mitochondrial ROS and succinate. Elevated ROS levels can have detrimental consequences on cellular function, leading to DNA damage and the alteration of crucial cellular processes associated with oncogenesis. Furthermore, the accumulation of ROS and succinate has been shown to enhance the activity of the hypoxia-inducible factor 1 (HIF-1) transcription factor [13–16]. The transcriptional activation of target genes by the HIF-1 complex enables cancer cells to adapt to the hypoxic tumor microenvironment, promoting their survival and proliferation. In addition, HIF-1 influences the expression of genes involved in cell survival, apoptosis resistance, and other processes contributing to the aggressive behavior of cancer [17–19].

The HIF-1 transcription factor is a heterodimeric complex composed of HIF-1α and HIF-1β. Under normal circumstances, HIF-1β is stably expressed, while HIF-1α is continuously synthesized but rapidly degraded. This rapid turnover of HIF-1α ensures that its levels remain low in normal cells. The turnover process of HIF-1α involves prolyl hydroxylase (PHD), an enzyme responsible for hydroxylating specific proline residues on HIF-1α and marking HIF-1α for recognition by the von Hippel-Lindau tumor suppressor protein (pVHL), leading to its ubiquitination by the pVHL-E3 ligase complex for proteolytic degradation [20–23]. In certain cellular contexts, such as cancer cells, the accumulation of ROS and succinate resulting from dysregulation of mitochondrial metabolism can inhibit the activity of PHD, consequently increasing the stability of the HIF-1α protein. Additionally, ROS can increase the activity of HIF-1 by enhancing the transcription of HIF-1α and facilitating the translocation of HIF-1α to the nucleus [16, 24–26].

PDZ and LIM domain 2 (PDLIM2) is a protein that contains both PDZ and LIM domains, which are protein interaction modules shared by members of the PDLIM protein family for protein–protein interactions to form protein complexes [27]. PDLIM2 has been shown to regulate the stability and activity of different transcription factors, and plays a role in various biological processes, including inflammation, immunity, and cancer. It has been identified as a tumor suppressor gene in certain cancers. The functional mechanisms involve the inhibition of cell growth and proliferation and the promotion of apoptosis in cancer cells by regulating the activity of oncogenes and tumor suppressor genes, as well as modulating signaling pathways involved in inflammation, cell cycle control, and apoptosis. However, PDLIM2 has also been reported as a tumor-promoting gene in certain cancer types. The exact mechanisms by which PDLIM2 carries out its biological functions are still under study, and further research is needed to fully understand its regulatory roles in different cellular processes [28–32].

Globally, cancer incidence and mortality rates are continuously increasing, with lung cancer being one of the most commonly diagnosed form and the primary cause of cancer-related deaths. Therapeutic options for lung cancer treatment include surgery, radiation therapy, chemotherapy, and targeted drug therapy. However, medical treatment often leads to the development of resistance, resulting to relapse. Additionally, the survival rate of metastatic lung cancer is low. Therefore, there is a critical need to further explore the pathogenic mechanisms of lung cancer to develop more effective diagnostic and therapeutic strategies [33–35]. In this study, we investigated the role and functional mechanisms of PDLIM2 in the growth of lung cancer. We found that the gene expression of PDLIM2 was downregulated in lung cancer and correlated with a poor prognosis in lung cancer patients. Downregulation of PDLIM2 in lung cancer cells was highly associated with increased NF-κB activation and dysregulated expression of genes for mitochondrial function. Further studies revealed that the tumor-promoting effect of PDLIM2 downregulation in lung cancer involves the accumulation of ROS, succinate, and the associated activation of HIF-1α.

## Materials and methods

### Reagents and antibodies

PX-478 was obtained from Selleckchem (TX, USA). MitoSOX, MitoTracker Green FM, and MitoTracker Red CMXRos were purchased from Molecular Probes/Invitrogen (MA, USA). BMS-345541, Hoechst 33,342, hydrogen peroxide, succinate, and dimethyl succinate were obtained from Sigma-Aldrich (MO, USA). Human and mouse recombinant TNF-α and IL-1β were purchased from Peprotech (NJ, USA). The following antibodies were used: Anti-SDHA, HIF-1α, PHD-2 from Cell Signaling Technology (MA, USA); anti-SDHB, SDHC, and SDHD from Abcam (Cambridge, UK); anti-mouse PDLIM2 from Invitrogen (MA, USA); anti-human PDLIM2 from OriGene (MD, USA); anti-β-actin from Novus Biologicals (CO, USA).

### Cell culture and stable transfection

Murine Lewis lung carcinoma (LLC) cells were cultured in Dulbecco's modified Eagle medium (DMEM). Human A549 lung cancer cells were maintained in Roswell Park Memorial Institute (RPMI) 1640 medium. Both basal culture media were supplemented with 10% fetal bovine serum (FBS), 100 U/ml penicillin, 100 μg/ml streptomycin, 0.25 µg/mL amphotericin B, 2 mM L-glutamine, and 10 mM HEPES. Short hairpin RNA (shRNA) constructs targeting PDLIM2 were purchased from the RNAi core facility (Academia Sinica, Taipei, Taiwan). Lentiviruses were generated by transfecting lentiviral vectors and packaging plasmids into 293T cells using the TransIT-X2 Dynamic Delivery System (Mirus Bio, WI, USA). Cancer cells were infected in the presence of viral supernatants and 8 μg/ml polybrene, and stable cell lines were selected using puromycin (3 ng/ml).

### Bioinformatics analysis

The UALCAN database (https://ualcan.path.uab.edu/) [36] was analyzed for the expression levels of PDLIM2 and HIF-1α in lung adenocarcinoma patients at different stages and their prognosis according to the Kaplan–Meier plotter estimates (https://kmplot.com/analysis/) [37]. Co-expression of PDLIM2 and HIF-1α were analyzed in lung adenocarcinoma cancer genomics using the public database cBioportal (https://www.cbioportal.org/) [38].

### Microarray analysis

The gene expression profiles in the PDLIM2 knockdown LLC cells and their corresponding control cells was analyzed using the Affymetrix Clariom S Assay (Affymetrix, CA, USA). The microarray experiment was conducted at the Microarray Core Laboratory in the National Health Research Institute (Miaoli, Taiwan). The microarray data generated in this study were deposited in the NCBI GEO database under the accession numbers GSE256236.

### RNA isolation, reverse transcription-quantitative PCR, and cancer tissue analysis

Total RNA was extracted from cancer cells and tumors using the illustra RNAspin Mini Kit (Cytiva, MA, USA) and TRIzol reagent (Invitrogen, MA, USA), respectively, following the manufacturer’s protocols. First-strand cDNA was synthesized from total RNA using the SuperScript IV First-Strand Synthesis System with random primers (Invitrogen, MA, USA). Quantitative PCR was performed with the QuantiNova SYBR Green PCR Kit (Qiagen, MD, USA) using the Applied Biosystems ViiA 7 Real-Time PCR System (Applied Biosystems, CA, USA) with gene-specific primers (Supplementary Table S1) for gene expression analysis. For human cancer tissue analysis, Cancer Survey cDNA Array 96-I, Lung Cancer cDNA Array III, and Lung Cancer cDNA Array V (OriGene, MD, USA) were used to analyze expression of PDLIM2 and HIF-1α genes. All primers used were synthesized by Protech Technology (Taipei, Taiwan). The relative expression of target gene was calculated using the comparative ΔΔ cycle threshold (Ct) method and normalization to β-actin expression.

### Western blot analysis

Total protein extracts from cancer cells and tumors were prepared using M-PER reagent (Thermo Scientific, MA, USA) containing a protease inhibitor cocktail (Thermo Scientific, MA, USA). An equal amount of protein was subjected to electrophoresis on a 10% SDS-PAGE gel and then transferred onto a polyvinylidene fluoride (PVDF) membrane (Millipore, MA, USA). After blocking non-specific protein binding, the membranes were incubated with specific antibodies as indicated, followed by incubation with horseradish peroxidase-conjugated secondary antibodies. Immunoreactive signals were visualized using a chemiluminescence (ECL) detection system (Millipore, MA, USA). The intensity of immunoreactive bands was quantified using ImageJ software, and β-actin expression was used as a loading control for normalization.

### Animal experiments

C57BL/6J mice were purchased from the National Laboratory Animal Center (Taipei, Taiwan). The mice were housed at the Laboratory Animal Center of the National Health Research Institutes (NHRI). All procedures were approved by the Institutional Animal Care and Use Committee (IACUC) of the NHRI. The mice used in this study were 6–8 weeks of age and were maintained and handled following the guidelines. shEmpty and shPDLIM2 stably expressed LLC cells were suspended in PBS, and mixed with Matrigel matrix (Corning, NY, USA) at a 1:1 ratio to a total volume of 100 μl. The cells were then subcutaneously injected into the mice. When the tumor size reached approximately 300 mm^3^, the mice were orally administered PX-478 three times per week for 2 weeks. Tumor volume was measured using the following formula: Tumor volume (mm^3^) = (length × width^2^)/2.

### Oxygen consumption measurements

The oxygen consumption rate (OCR) was measured using the Seahorse XFe24 Analyzer (Seahorse Bioscience, MA, USA). Briefly, cell monolayers were cultured in XF Cell Culture Microplates (Seahorse Bioscience) at a density of 2.5 × 10^4^ cells/well (for LLC cells) or 5 × 10^4^ cells/well (for A549 cells). After 16 h, the growth medium was replaced with XF assay medium (XF base medium supplemented with 1% FBS, 2 mM L-glutamine, 1 mM HEPES, and 25 mM glucose), adjusted to pH 7.4. The cells were then incubated for 1 h at 37 °C without CO_2_. The compounds were injected sequentially as follows: 1 mM oligomycin; 0.5 mM FCCP; 0.5 mM rotenone/antimycin A (from Seahorse XF Cell Mito Stress Test Kit, Seahorse Bioscience). The protocol and algorithm for OCR responses were analyzed using Wave 2.4 software (Seahorse Bioscience).

### Metabolite extraction from cancer cells

The cell pellets were washed three times with PBS and then suspended in 1 ml of ice-cold 100% methanol. These suspensions were snap-frozen in liquid nitrogen for preservation. Upon thawing, the cell pellets were vortexed for 30–60 s and subsequently centrifuged at 800 × g for 1–2 min. The supernatants were carefully transferred to a new microcentrifuge tube and kept on ice. The pellets obtained from the previous step were resuspended in 1 ml of ice-cold 100% methanol and snap-frozen again. Following another round of thawing, vortexing, and centrifugation, the supernatants were combined with the initially harvested ones. The combined supernatants were then dried using a centrifugal evaporator to remove the solvent. After complete drying, the resulting dried metabolite pellets were resuspended in H_2_O and subsequently subjected to LC–MS analyses.

### Metabolomic analysis

The TCA cycle metabolism products from LLC and A549 cells were analyzed using liquid chromatography-mass spectrometry (LC–MS). The Acquity ultraperformance liquid chromatography (UPLC)-Xevo TQ-XS System (Waters Corporation) with an ACQUITY BEH C18 column (2.1 mm × 100 mm × 1.7 mm, Waters Corporation) was used. The liquid chromatography separation was carried out at 45 °C with a flow rate of 0.3 mL/min using the following gradient of elute A and B for the analysis: 0–2.5 min 1% B, 2.5–4.5 min from 5% B to 100% B, 4.5–5 min 100% B, 5–9 min from 100 to 1% B (elute A: water with 10 mM tributylamine and 15 mM acetic acid; elute B: 50% acetonitrile with 10 mM tributylamine and 15 mM acetic acid). The column was then re-equilibrated for 3 min. Mass was operated in negative mode and positive mode with multiple reaction monitoring. The capillary voltage was maintained at 1000 V, and the desolvation and source temperature at 500 °C and 150 °C, respectively. Desolvation gas flow was set at 1000 L/h.

### Flow cytometry

Mitochondrial reactive oxygen species (ROS) levels were detected by staining cells with 5 μM MitoSOX for 30 min at 37 °C. To measure mitochondrial mass, cells were incubated with MitoTracker Green FM and MitoTracker Red CMXRos at 100 nM for 30 min at 37 °C. For intracellular staining, cells were fixed with the Transcription Factor Buffer Set according to the manufacturer's instructions (BD Biosciences). The cells were then intracellularly stained with Alexa Fluor 647-Phospho-NF-κB p65 (93H1, Cell Signaling) for 50 min. Cells were then washed with FACS buffer, and fluorescence was analyzed using a FACSCanto II Flow Cytometer (BD Biosciences). Data were analyzed using FlowJo software.

### Mitochondrial morphology analysis

Cells were seeded in a glass-bottom dish (35 mm) at low density, and live cells were stained with 100 nM MitoTracker Green FM and 5 μg/ml Hoechst 33,342 for 30 min at 37 °C. After three washes, the cells were visualized under a confocal microscope (Leica TSC SP5).

### Immunohistochemistry

Paraffin-embedded tumors were sectioned into 5 μm tissue slides. These tissue slides were rehydrated with graded concentrations of ethanol to PBS, and endogenous peroxidase was blocked with 3% hydrogen peroxide for 5 min. For HIF-1α staining, the primary antibody against HIF-1α was used at a 1:25 dilution and incubated at room temperature for 1 h. The tissue sections were then incubated with horseradish peroxidase-conjugated secondary antibody at room temperature for 30 min following washing with PBST. The detection was processed with the Discovery XT automated IHC/ISH slide staining system (Ventana Medical System, Inc., Tucson) using an ultraView Universal DAB Detection Kit (Ventana Medical System, Inc., Tucson), according to the manufacturer's instructions. Immunostaining was visualized following hematoxylin counterstaining. HIF-1α-positive area scores and leukocyte infiltration were evaluated using ImageJ software.

### Statistical analysis

Data are presented as the mean ± SEM from at least three independent experiments for cell studies and at least six mice per group for animal studies. Statistical significance was determined by a two-tailed Student’s t-test. A value of *p* < 0.05 was considered statistically significant. Graphs were prepared using GraphPad Prism (GraphPad Software v8.0.1).

## Results

### Low expression of PDLIM2 is associated with a poor prognosis in lung cancer

**Fig. 1:**
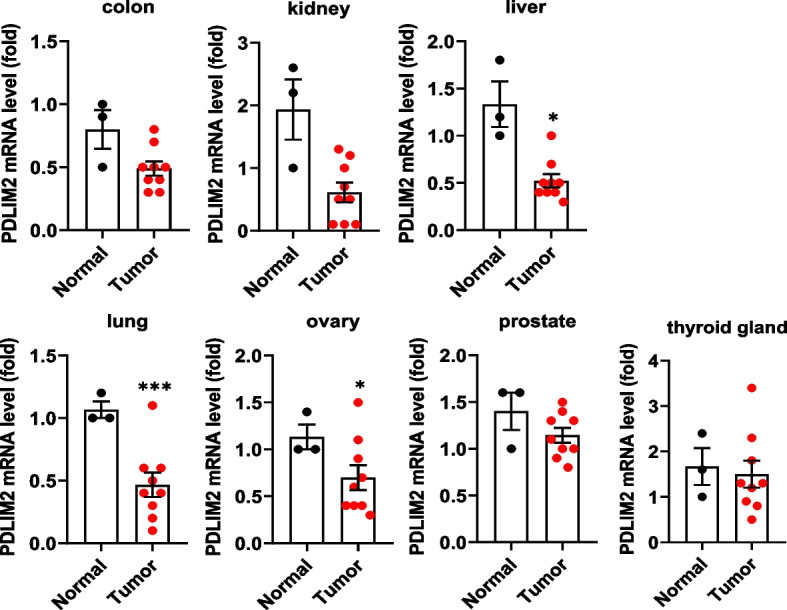
PDLIM2 expression in human cancers. Real-time PCR analysis of PDLIM2 levels was performed using a TissueScan cancer survey panel, including tissues from colon, kidney, liver, lung, ovary, prostate, thyroid gland tumors, and their corresponding non-tumor tissues. The expression levels were normalized to β-actin mRNA levels. Data represent means ± SEM. * *p* < 0.05, ** *p* < 0.01, *** *p* < 0.001 compared with the normal tissues

**Fig. 2:**
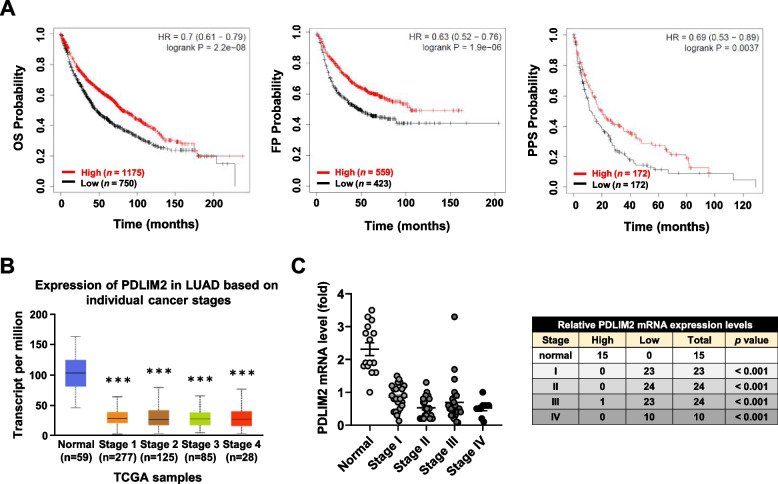
Significance of PDLIM2 expression in lung cancer pathogenesis and clinical outcomes. **A** Kaplan–Meier survival curve depicting the overall survival (OS), first progression (FP), and post-progression survival (PPS) of lung cancer patients (data sourced from Kaplan–Meier Plotter). **B** Expression levels of PDLIM2 in normal tissue compared to different stages of lung adenocarcinoma from patients, utilizing the TCGA dataset. Data and *p*-values (*** *p* < 0.001) are provided by the UALCAN database. **C** Analysis of PDLIM2 expression in TissueScan lung cancer cDNA arrays, comparing normal tissue (*n* = 15) and tumor biopsies (*n* = 81) across various tumor stages

To assess the role of PDLIM2 in cancer growth, we screened a human TissueScan cancer survey panel for the expression of PDLIM2 mRNA in various types of cancers. This panel included cDNA samples prepared from both normal and cancer tissues of patients with different types of cancers. Each set of cDNA samples contained 9 samples from individual patients’ cancer tissue and 3 from normal tissue. As shown in Fig. 1, the mRNA level of PDLIM2 was significantly decreased in liver, lung, and ovary cancer compared to non-tumor tissues. In this study, we focused on lung cancer to investigate the function and mechanism of PDLIM2 in tumor growth as lung cancer is the leading cause of cancer-related deaths, and there is an unmet need for its treatment. We further examined the correlation between PDLIM2 gene expression and the clinical prognosis of lung cancer patients. The Kaplan–Meier plotter demonstrated that low expression of PDLIM2 was associated with poor overall survival (OS), first progression (FP), and post-progression survival (PPS) of patients (Fig. 2A). An analysis of TCGA data related to PDLIM2 in lung cancer using the UALCAN database revealed a significant decrease in PDLIM2 mRNA expression in patients at different stages compared to normal tissue (Fig. 2B). Similarly, results from TissueScan lung cancer cDNA arrays indicated downregulation of PDLIM2 expression in tumor tissues from stages I to IV patients (Fig. 2C). Collectively, these findings demonstrate that reduced expression of PDLIM2 is associated with lung cancer progression.

### Knockdown of PDLIM2 in cancer cells impairs the expression of genes related to mitochondria metabolism and promotes tumor growth

**Fig. 3:**
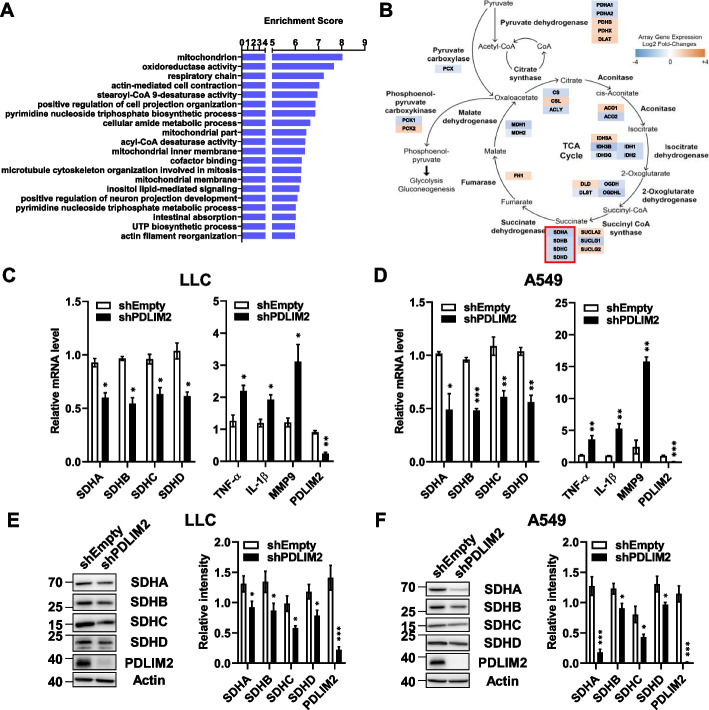
PDLIM2 downregulation impairs expression of genes for mitochondrial functions. **A** Gene ontology enrichment analysis of microarray data by comparing shEmpty and shPDLIM2 LLC cells. Analysis focused on changes with -log fold-changes exceeding 2 or falling below -2. **B** Fold change in the expression of TCA cycle-related genes derived from the mitochondrial term in microarray analysis. The genes encoding subunits of SDH complex are boxed with red color. **C**,** D** mRNA expression levels of SDH subunits, TNF-α, IL-1β, and MMP9 were measured by real-time PCR following stable PDLIM2 knockdown in LLC and A549 cells. **E**,** F** Protein levels of SDH subunits in PDLIM2 knockdown LLC and A549 cells were measured by immunoblotting. Data represent means ± SEM. * *p* < 0.05, ** *p* < 0.01, *** *p* < 0.001 compared to the shEmpty group

To investigate the functions of PDLIM2 in lung cancer cells, we established stable PDLIM2 knockdown LLC and A549 cell lines through the transfection of PDLIM2-targeted shRNAs. We used shPDLIM2#840 LLC cells (referred to as shPDLIM2 LLC) and shPDLIM2#147 A549 cells (referred to as shPDLIM2 A549) for further studies due to their efficient PDLIM2 knockdown (Supplementary Fig. S1). We performed DNA microarray analysis to assess gene expressions in shPDLIM2 LLC cells and their corresponding control (shEmpty) cells. Gene ontology enrichment analysis revealed that PDLIM2 highly regulates genes associated with mitochondria function, oxidoreductase activity, and respiratory chain (Fig. 3A). The TCA cycle is a central hub for biosynthesis and metabolism within mitochondria. Through oxidoreductase reactions, it is linked to both complex I and complex II of the respiratory chain at the inner mitochondrial membrane, driving energy production. Mutation in genes encoding enzymes in TCA cycle, particularly those located at the points where the TCA cycle and respiratory chain coupled, have been demonstrated to result in the accumulation of oncometabolites in cancer cells [6, 39–41]. These findings led us to investigate the role of TCA genes in mediating the functions of PDLIM2. The microarray data revealed varying expression profiles for genes in different TCA enzyme complexes in shPDLIM2 LLC cells, with some upregulated and others downregulated. However, all genes encoding subunits of the SDH complex were downregulated (Fig. 3B). The SDH complex contains four subunits and is also known as the complex II of the respiratory chain [13, 14]. Downregulation of the SDHA, SDHB, SDHC, and SDHD expressions in shPDLIM2 LLC cells were further confirmed with RT-qPCR (Fig. 3C, left panel). Similar downregulation of these SDH genes was also observed in shPDLIM2 A549 cells (Fig. 3D, left panel). Given that a reduction in PDLIM2 expression is known to increase NF-κB activity and the expression of inflammatory genes [28, 32], we also assessed the expression of TNF-α, IL-1β and MMP9 in the PDLIM2 knockdown cells for comparison. As expected, these inflammatory genes were upregulated in both the shPDLIM2 LLC and shPDLIM2 A549 cells (Fig. 3C and D, right panel). Consistent with the decreased mRNA expressions, the protein levels of these SDH genes were also reduced in shPDLIM2 LLC and shPDLIM2 A549 lung cancer cells (Fig. 3E and F).

The molecular mechanism underlying the downregulation of SDH genes in PDLIM2 knockdown cells was investigated. We explored the regulation of SDH genes by NF-κB activity. Our previous study has demonstrated the induction of NF-κB activations by TNF-α and IL-1β stimulations, with NF-κB activations being inhibited by PDLIM2 expression [42]. In the current study, we observed increased NF-κB activity in PDLIM2 knockdown LLC and A549 lung cancer cells (Supplementary Fig. S2A and B). Treatment of these cells with TNF-α and IL-1β suppressed the expression of the four SDH genes, and this suppression was reversed by treatment with a NF-κB inhibitor, BMS-345541 [43] (Supplementary Fig. S2C and D). These results are consistent with previous reports indicating that NF-κB suppresses the expression of SDH genes [44–46].

**Fig. 4:**
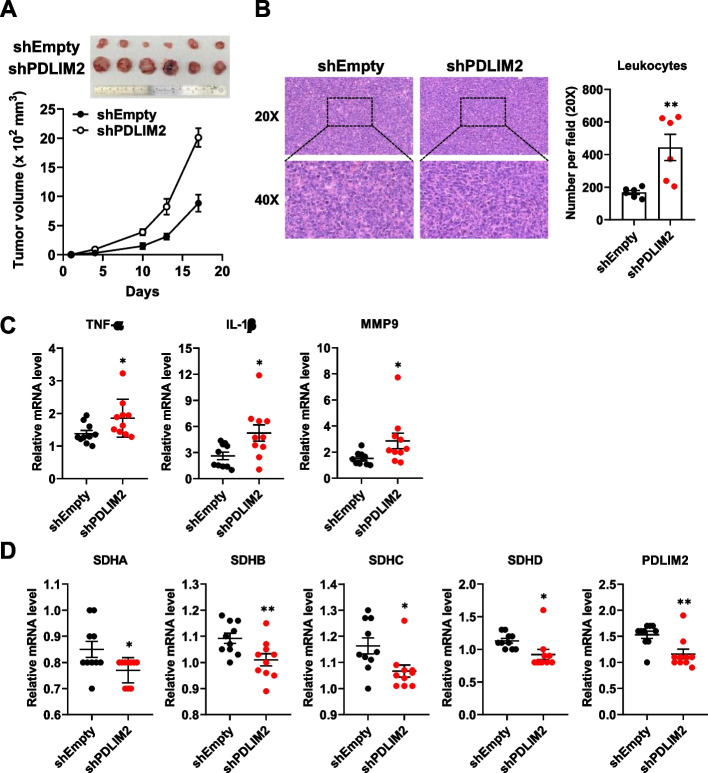
PDLIM2 downregulation promotes tumor growth and suppresses SDH gene expression. **A** C57BL/6 mice were injected subcutaneously with 5 × 10^5^ control and PDLIM2 knockdown LLC cells. Tumor volume was measured at the indicated time points, and the mean tumor size was plotted. Mice were euthanized on day 17, and tumors were excised and photographed. **B** H&E staining of tumor samples to visualize leukocyte infiltrations (left upper panel: 20X, left lower panel: 40X). Leukocyte infiltrations in 20X magnification areas were quantified using ImageJ software (Right panel). **C**,** D** Total RNAs were isolated from the tumors using TRIzol reagent. mRNA expression levels of TNF-α, IL-1β, MMP9, and SDH subunits were measured by real-time PCR. The expression levels were normalized to β-actin mRNA levels. Data represent means ± SEM. * *p* < 0.05, ** *p* < 0.01 compared with the shEmpty group

To further investigate the impact of PDLIM2 downregulation on tumor growth and the expression of TCA cycle-related genes within tumors, we utilized an allograft mouse model for study. Mice were inoculated with PDLIM2 knockdown and their corresponding control LLC cells. Tumors derived from the PDLIM2 knockdown cells exhibited a more rapid growth rate (Fig. 4A). Similar to the results observed in shPDLIM2 LLC cells, these tumors displayed signs of inflammation, including leukocyte accumulation and increased expression of inflammatory cytokines (Fig. 4B and C), and showed lower mRNA expression levels of SDHA, SDHB, SDHC, and SDHD (Fig. 4D). These findings indicate that PDLIM2 deletion elevates NF-κB activity and impairs the expression of mitochondrial SDH genes and is associated with increased tumor growth.

### Downregulation of PDLIM2 causes mitochondrial dysfunction in lung cancer cells

**Fig. 5:**
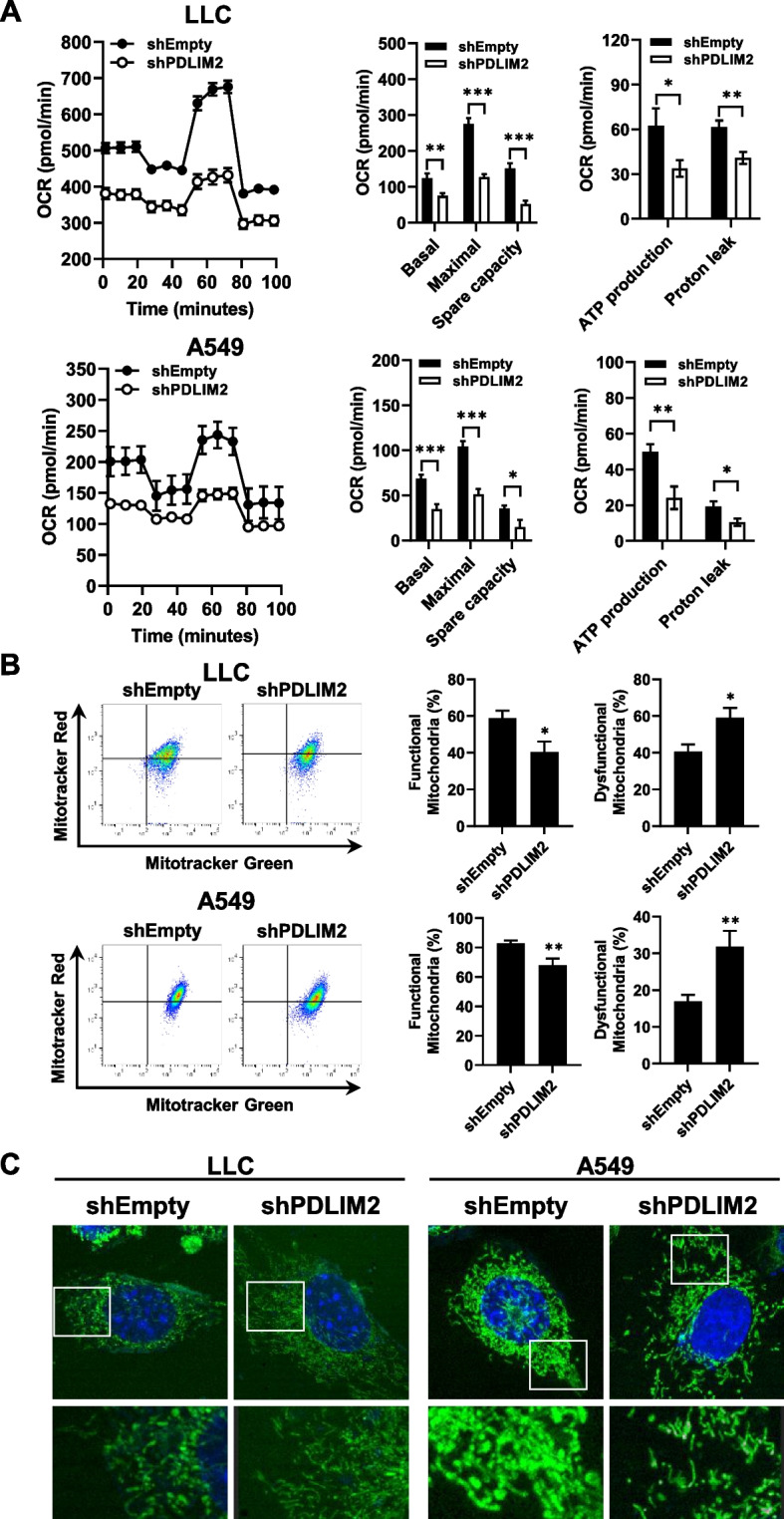
PDLIM2 knockdown impairs mitochondrial functions in lung cancer cells. **A** Real-time measurement of cellular oxygen consumption rate (OCR) in control and PDLIM2 knockdown LLC and A549 lung cancer cells using the Seahorse XF24 Extracellular Flux Analyzer. Basal respiration, maximal respiration, spare capacity, ATP production, and proton leak were assessed, respectively. **B** Accumulation of dysfunctional mitochondria in control and PDLIM2 knockdown LLC and A549 lung cancer cells revealed by staining with MitoTracker Green and MitoTracker Red. Representative dot plots from FACS analysis are shown. The comparison between MitoTracker Green^+^ Red^+^ (functional) and MitoTracker Green^+^ Red^−^ (dysfunctional) populations is presented. Data represent means ± SEM. * *p* < 0.05, ** *p* < 0.01, *** *p* < 0.001 compared with the shEmpty group. **C** Confocal microscopy images depict the mitochondrial morphology of control and PDLIM2 knockdown LLC and A549 cells. Green fluorescence represents mitochondria, and blue fluorescence represents Hoechst-labeled nucleus

In light of the observed changes in the expression of TCA cycle-related genes and the decreased expression of SDH genes in PDLIM2-knockdown cells and tumors (Figs. 3 and 4), we conducted further investigations to determine whether PDLIM2 downregulation affected mitochondrial function. We assessed mitochondrial respiration in both control and PDLIM2-knockdown lung cancer cells by measuring the oxygen consumption rate (OCR) using the Seahorse Mito Stress assay. Major parameters of the OCR, including basal respiration, maximal respiration, spare respiratory capacity, ATP production, and proton leak, were all reduced in PDLIM2-knockdown LLC cells and A549 cells (Fig. 5A). These findings indicate that mitochondrial respiration was impaired in the PDLIM2-knockdown lung cancer cells. Additionally, we employed two types of mitochondria-specific dyes to distinguish respiring mitochondria (MitoTracker Red) and total mitochondria (MitoTracker Green) through FACS analysis. The results revealed an increased population of dysfunctional mitochondria (MitoTracker Green-positive, MitoTracker Red-negative) in PDLIM2-deficient lung cancer cells (Fig. 5B). The tubular morphology of mitochondria is dynamically changed through a balanced process of fusion and fission in response to the metabolic alterations. Mitochondrial fission disrupts the integrity of mitochondria, leading to their dysfunction [47, 48]. Following MitoTracker Green and Hoechst staining, the morphology of mitochondria in cells was observed with confocal microscopy. The results indicated that mitochondrial fission was increased in the PDLIM2-knockdown LLC cells and A549 cells (Fig. 5C). These findings suggest that the altered expression of TCA cycle-related genes and the reduced expression of SDH genes in PDLIM2-knockdown cells are associated with mitochondrial dysfunction.

### Downregulation of PDLIM2 leads to accumulation of mitochondrial reactive oxygen species and oncometabolites in lung cancer cells

**Fig. 6:**
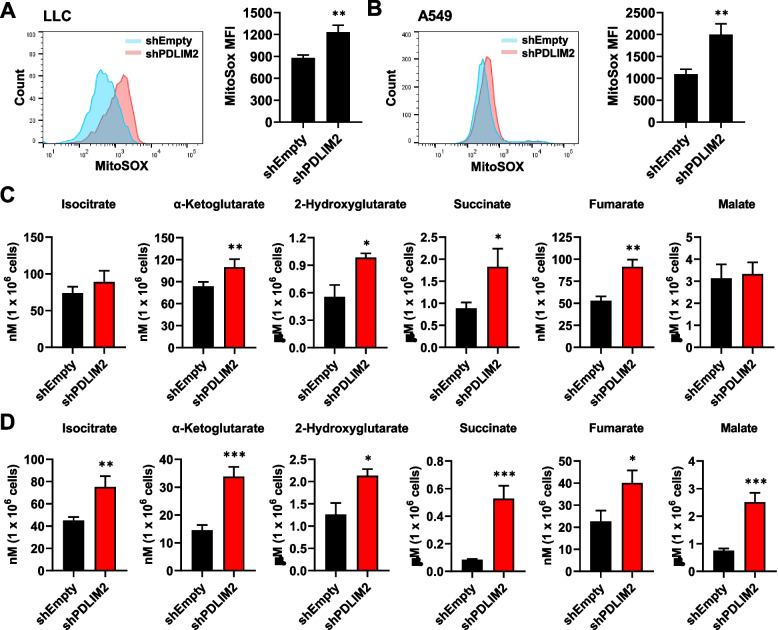
Increased accumulation of mitochondrial ROS and oncometabolites in PDLIM2 knockdown lung cancer cells. **A**,** B** Mitochondrial ROS in control and PDLIM2 knockdown LLC (**A**) and A549 (**B**) lung cancer cells was analyzed by flow cytometry using the MitoSOX Red probe. **C**,** D** Analysis of metabolite levels in PDLIM2 knockdown LLC (**C**), A549 (**D**) cells and their corresponding controls by LC–MS. Data represent means ± SEM. * *p* < 0.05, ** *p* < 0.01, *** *p* < 0.001 compared with the shEmpty group

The accumulation of mitochondrial reactive oxygen species (mtROS) and oncometabolites such as succinate has been demonstrated in mitochondrial fission and dysregulated mitochondria, contributing to promoting tumor growth [13–15, 47–50]. We performed MitoSOX staining to analyze mtROS production. PDLIM2-knockdown LLC and A549 cells exhibited a significant increase in MitoSOX fluorescence intensity, indicating the accumulation of mtROS (Fig. 6A and B). To further investigate whether the succinate and other metabolites within the TCA cycle were accumulated due to the observed altered regulation of genes for SDHs and other enzymes in the cycle (as shown in Fig. 3), we conducted liquid chromatography-mass spectrometry (LC–MS) analysis to measure the levels of various metabolites in PDLIM2-knockdown lung cancer cells. The results indicated a substantial increase in α-ketoglutarate, 2-hydroxyglutarate, succinate, and fumarate levels in PDLIM2-knockdown LLC cells (Fig. 6C). Additionally, PDLIM2 knockdown in A549 cells also led to increased levels of isocitrate and malate (Fig. 6D). Taken Together, these findings suggest that the alteration in TCA cycle-related genes resulting from PDLIM2 downregulation leads to the accumulation of mtROS, and oncometabolites including succinate in lung cancer cells.

### Downregulation of PDLIM2 is associated with increased expression of HIF-1α in lung cancer

**Fig. 7:**
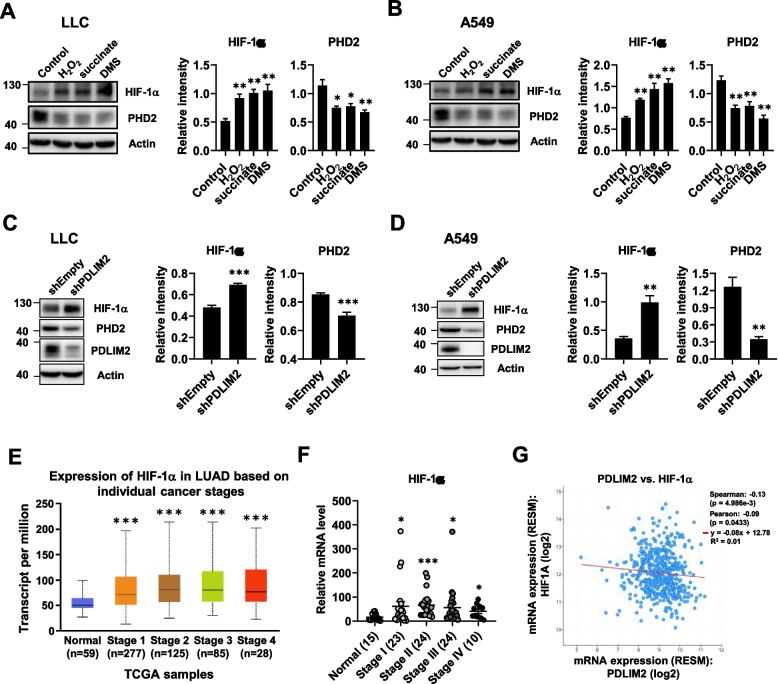
Downregulation of PDLIM2 in lung cancer is associated with increased HIF-1α expression. **A**, **B** LLC and A549 lung cancer cells were treated with 400 μM H_2_O_2_, 5 mM succinate, and 20 mM dimethyl succinate (DMS) for 6 h. Protein level of HIF-1α and PHD2 was measured by immunoblotting. **C**,** D** Protein level of HIF-1α and PHD2 was measured by immunoblotting in PDLIM2 knockdown LLC and A549 cells. **E** UALCAN database provided data showing HIF-1α expression in normal and different stages of lung adenocarcinoma from patients in the TCGA dataset, with corresponding *p*-values (*** *p* < 0.001). **F** HIF-1α expression was analyzed in TissueScan lung cancer cDNA arrays with normal tissue (*n* = 15) and tumor biopsies (*n* = 81) across various tumor stages. **G** Co-expression analysis of PDLIM2 and HIF-1α by using cBioportal for cancer genomics. * *p* < 0.05, ** *p* < 0.01, *** *p* < 0.001 compared with the shEmpty group, control, and the normal tissue, respectively

Reactive oxygen species and succinate have been shown to increase the stabilization and activation of hypoxia-inducible factor 1α (HIF-1α) by inactivating prolyl hydroxylases (PHD) [15, 16, 24, 25]. Of the PHD isoforms (PHD1-3), PHD2 is the major regulator of HIF-1α [51, 52]. To verify the effect of ROS and succinate on HIF-1α and PHD2 expression in lung cancer cells, we aimed to increase the levels of ROS and succinate within the cells. LLC and A549 lung cancer cells were treated with hydrogen peroxide (H_2_O_2_) and MitoSOX staining was performed to analyze mtROS level. Both LLC and A549 cells exhibited a significant increase in mtROS accumulation (Supplementary Fig. S3A and B). Additionally, the intracellular levels of succinate in LLC and A549 cells were increased by treatment with succinate and dimethyl succinate, as confirmed by LC–MS analysis (Supplementary Fig. S3C and D). H_2_O_2_ is a common ROS inducer [53]. Dimethyl succinate is modified from succinate for better cell permeability. Both succinate and dimethyl succinate were used to treat cells to increase intracellular level of succinate [54]. These increased levels of ROS and succinate downregulated the protein expression level of PHD2 and increased the level of HIF-1α in lung cancer cells (Fig. 7A and B). The results are consistent with previous reports indicating that ROS and succinate negatively and positively regulate of PHD2 and HIF-1α expression, respectively [15, 16, 24, 25]. Since knockdown of PDLIM2 leads to the accumulation of mtROS and succinate (Fig. 6), we further investigated the protein levels of HIF-1α and PHD2 in PDLIM2 knockdown lung cancer cells. The results indicated that the downregulation of PDLIM2 reduced the protein expression level of PHD2 and increased the level of HIF-1α in LLC and A549 cells (Fig. 7C and D). These results suggest that HIF-1α activity is upregulated in PDLIM2 knockdown lung cancer cells via the intracellular ROS and succinate regulated PHD2 downregulation. Furthermore, it has been demonstrated that ROS can increase the gene transcription of HIF-1α [26, 55, 56]. Therefore, we also investigated whether PDLIM2 regulates the HIF-1α expression in lung cancer. The expression level of HIF-1α in patients with lung cancer was evaluated using the UALCAN database and TissueScan lung cancer cDNA arrays. The results revealed a significant increase in HIF-1α mRNA expression in patients at different stages compared with normal tissue (Fig. 7E and F). Additionally, analyzing the TCGA dataset using cBioportal for cancer genomics showed a reverse correlation between PDLIM2 and HIF-1α in lung cancer (Fig. 7G). In summary, these findings suggest that HIF-1α is negatively regulated by PDLIM2 in lung cancer.

### Inhibition of PDLIM2-knockdown promoted tumor growth by HIF-1α inhibitor

**Fig. 8:**
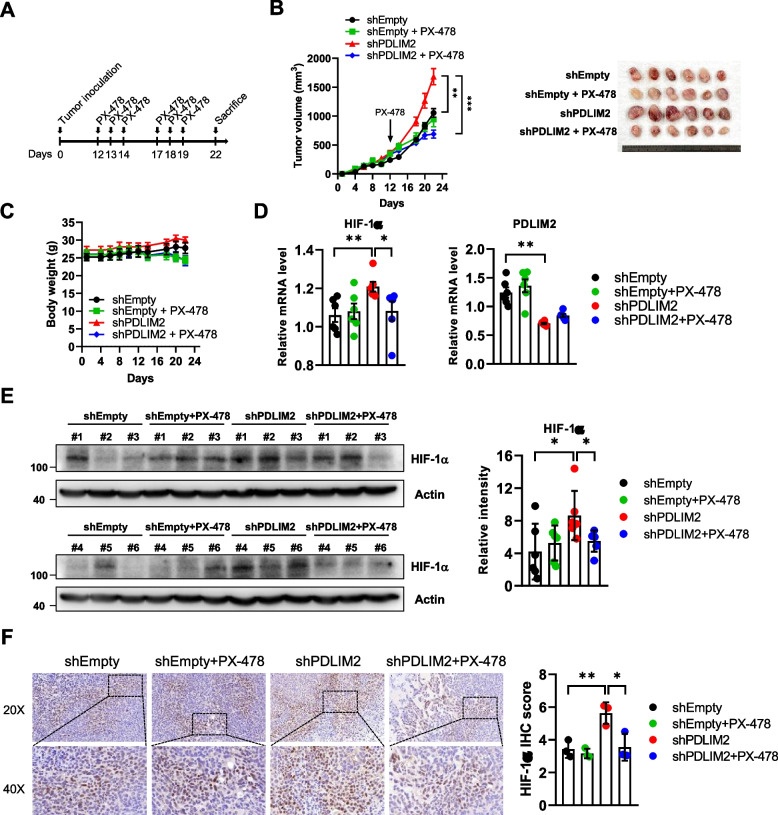
HIF-1α inhibitor abolishes the tumor-promoting effects of PDLIM2 downregulation. **A** C57BL/6 mice (*n* = 6) were injected subcutaneously with 5 × 10^5^ control and PDLIM2 knockdown LLC cells. When the tumors reached approximately 300 mm^3^, the mice were orally administered PX-478 three times per week for 2 weeks. **B** Tumor volume was measured at the indicated times, and the mean tumor size was plotted. Mice were euthanized on day 22, and tumors were excised and photographed (right panel). **C** The body weight of each mouse was measured. **D** Total RNAs from the tumors were isolated using TRIzol reagent, and the mRNA expression levels of HIF-1α and PDLIM2 were measured by real-time PCR. The expression levels were normalized to β-actin mRNA levels. **E** Protein levels of HIF-1α were measured by immunoblotting in tumor tissues. **F** Immunohistochemistry of tumor samples for HIF-1α positive tumor cells (left upper panel: 20X, left lower panel: 40X). HIF-1α-positive area scores were evaluated by using ImageJ software at 20X magnification filed (Right panel). Data represent means ± SEM. * *p* < 0.05, ** *p* < 0.01, *** *p* < 0.001 compared with the shEmpty group

To further confirm the role of HIF-1α in promoting tumor growth under PDLIM2-knockdown conditions, LLC cancer animal models were established. Tumors were allowed to grow to approximately 300 mm^3^, and then the mice were orally administered with PX-478 three times per week for 2 weeks (Fig. 8A). PX-478 inhibits HIF-1α at multiple levels, including transcription and translation [57, 58]. As shown in Fig. 8B, PX-478 treatments significantly reduced the tumor growth promotion caused by PDLIM2 knockdown in LLC cells. However, these treatments had no effect on inhibiting tumors derived from shEmpty LLC cells. The body weights of mice in the different groups with and without PX-478 treatments did not show any significant difference during the experimental period (Fig. 8C). The connection between PDLIM2 and HIF-1α expressions in these tumors was further investigated. The mRNA expression level of HIF-1α was upregulated in tumors derived from shPDLIM2-knockdown LLC cells compared to the level in tumors derived from the shEmpty control cells. The upregulation of HIF-1α in shPDLIM2-knockdown tumors was inhibited by PX-478 treatment. In contrast, the HIF-1α level of shEmpty tumors was not affected by PX-478 treatment (Fig. 8D, left panel). The expression of PDLIM2 was also verified in these tumors, and the results showed that PX-478 had no effect on the PDLIM2 expression in both shPDLIM2 and shEmpty tumors (Fig. 8D, right panel). In addition, protein levels in tumor cell lysates and tumor tissues investigated by immunoblotting and IHC staining respectively, revealed an increase in HIF-1α level in tumors derived from PDLIM2 knockdown cells, and which was blocked by PX-478 (Fig. 8E and F).

**Fig. 9:**
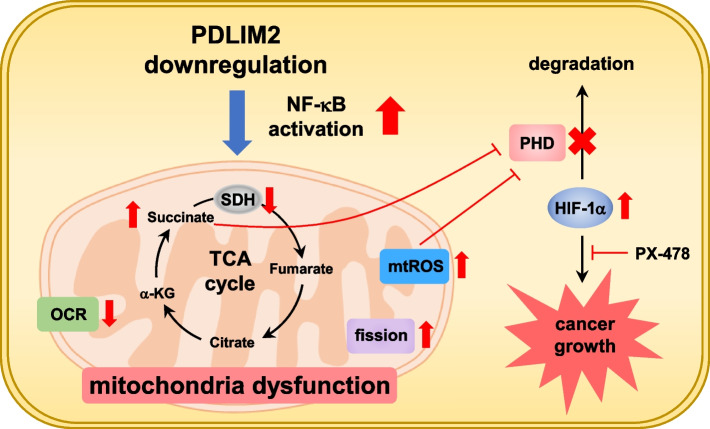
Illustrates a pro-tumor mechanism involving the activation of HIF-1α by PDLIM2 downregulation. In lung cancer, decreased PDLIM2 expression leads to NF-κB signaling activation, resulting in impaired SDH gene expression and mitochondrial dysfunction. This dysfunction includes reduced mitochondrial OCR, increased succinate accumulation, elevated mitochondrial fission, and ROS production. Succinate and ROS further inhibit PHD expression, leading to increased HIF-1α activation and ultimately promoting tumor growth. The inhibition of tumor growth by a HIF-1α inhibitor in this study suggests that HIF-1α may be a potential target for treating of cancers resulting from PDLIM2 downregulation

In summary, as depicted in Fig. 9, this study demonstrated that the downregulation of PDLIM2 leads to heightened NF-κB activity in lung cancer cells, thereby dysregulating SDH gene expression and mitochondrial functions. The increased accumulations of succinate and mtROS inactivate PHD, consequently upregulating HIF-1α, which significantly contributes to tumor growth promotion in PDLIM2-knockdown conditions. Therefore, HIF-1α inhibitors may be a potential strategy for treating cancers resulting from PDLIM2 downregulation (Fig. 9).

## Discussion

PDLIM2 has been reported to exert varying effects and employ diverse mechanisms in controlling tumor growth across different cancer types [30–32]. In ovarian cancer, downregulation of PDLIM2 promotes tumor growth by modulating NOS2-derived nitric oxide signaling and the recruiting M2 type macrophages [59]. Additionally, PDLIM2 attenuates the proliferation, migration, and invasion of ovarian cancer cells by inactivating the TGF-β/Smad pathway [60]. In hepatocellular carcinoma, PDLIM2 functions as a tumor suppressor by negatively regulating β-catenin [61]. In breast, colorectal, and lung cancers, PDLIM2 expression is epigenetically suppressed at the transcriptional level or regulated by microRNAs posttranscriptionally. Reduction of PDLIM2 in these cases results in the constitutive activation of transcription factors, including NF-κB and STAT3, leading to increased tumor inflammation and supporting tumor growth [62–66]. However, higher expression of PDLIM2 has also been observed in certain cancer types, such as triple-negative breast cancer, esophageal squamous cell carcinoma, acute myeloid leukemia, and prostate cancer, where it has been suggested as a prognostic marker [67–70]. Presently, there is no consensus on how PDLIM2 can act as a tumor suppressor in some cancer types while promoting tumor growth in others. These distinct effects of PDLIM2 on the regulation of tumor growth suggest its involvement in a more complex regulatory network than currently understood, highlighting the need for further research in this area.

Lung cancers is one of the most commonly diagnosed and deadliest forms of cancer, significantly impacting both individual patients and their families’ quality of life. Effectively addressing the challenges posed by lung cancer requires a comprehensive understanding of its pathogenic mechanisms to develop a multifaceted approach for prevention, early detection, and treatment [33–35]. In this study, we investigated the role and underlying mechanisms of PDLIM2 in regulating lung cancer growth. We observed a significant downregulation of PDLIM2 expression in lung cancer. This reduction in PDLIM2 expression was consistent across all stages of lung cancer, from stage I to stage IV. Notably, tumors derived from PDLIM2-knockdown lung cancer cells exhibited an accelerated growth rate and increased expression of inflammation cytokines controlled by NF-κB. These findings are consistent with previously reported roles of PDLIM2 in lung cancer [63]. However, this study also revealed and explored a unique function of PDLIM2 downregulation; the suppression of SDH genes through elevated NF-κB activity and the regulation of mitochondria function. This includes the accumulation of mitochondrial ROS, oncometabolites, and the activation of HIF-1α, all of which play a crucial role in tumor growth regulation.

PDLIM2 has been demonstrated to regulate different signaling pathways, including NF-κB, STAT3, NOS2, TGF-β/Smad, and β-catenin in cancer cells [28–32]. Despite these known connections, its association with mitochondrial functions has not been investigated. Upon analyzing gene expression profiles in parental and PDLIM2 knockdown lung cancer cells, we observed a significant impact of PDLIM2 downregulation on the expression of genes involved in mitochondrial functions. Consistently, PDLIM2 knockdown in lung cancer cells affected mitochondrial functions, as indicated by the reduction in all the major parameters of the OCR, diminished respiring mitochondria, and increased mitochondrial fission in the cells. The TCA cycle, a central hub of mitochondrial metabolism, plays a pivotal role in generating building blocks for sugar, lipid, and amino acid syntheses, while coupling with the mitochondrial respiratory chain. Comprising a series of reactions, each step of the TCA cycle is catalyzed by a distinct enzyme complex [39–41]. Our analysis further revealed that PDLIM2 knockdown in lung cancer cells altered the expression of genes across various enzyme complexes in the TCA cycle. Notably, the major impact appeared to be on the regulation of the SDH complex, where downregulation of PDLIM2 suppressed the expression of all four subunits of the SDH complex. Conversely, the expression of different components in other enzyme complexes showed both positive and negative regulation. The expression of SDH genes was suppressed by the elevated NF-κB activity in the PDLIM2 knockdown cells. This finding is consistent with previous reports showing that stress or inflammatory stimuli activate NF-κB in cells, resulting in the inhibition of SDH gene expression [44–46]. Among them, IL-1, a potent NF-κB activator, was shown to reduce SDHB expression through the upregulation of DNA methyltransferase 1, leading to the epigenetic suppression of the SDHB promoter [45].

The SDH complex, also referred to as complex II of the mitochondrial respiratory chain. This complex consists of four subunits: SDHA and SDHB, which are two catalytic subunits protruding into the mitochondria matrix, and SDHC and SDHD, which are two subunits anchoring SDHA and SDHB to the inner mitochondrial membrane where the respiratory chain reactions occur [71, 72]. Concomitant with the downregulation of SDH complex genes, the protein levels of the SDH subunits were diminished in the PDLIM2-downregulated lung cancer cells. The SDH complex is essential for the TCA cycle to oxidize succinate into fumarate. Loss-of-function mutations in SDH genes have been associated with the development of pheochromocytoma and paraganglioma [73, 74]. Furthermore, inactive mutations in SDH complex genes have been linked to other cancer types, including renal cell cancer, neuroblastomas, testicular seminoma, gastrointestinal stromal tumors, and thyroid tumors [75–79]. These findings suggest that dysregulation of mitochondrial functions, the TCA cycle, and the SDH complex may contribute to the pro-tumoral effect of PDLIM2 downregulation. Consistently, PDLIM2 knockdown cells exhibited an accelerated tumor growth rate in mice. Tumors derived from these cells also displaying reduced expression of SDH genes.

Inactive mutations of SDH complex genes and dysregulation of mitochondrial function have been demonstrated to lead to the accumulation of ROS and succinate [80, 81]. Consistently, elevated levels of ROS and succinate were detected in PDLIM2-knockdown lung cancer cells. Additionally, increased levels of other metabolites derived from the TCA cycle, such as 2-hydroxyglutarate and fumarate, were also observed in the PDLIM2-reduced cells. The accumulation of these metabolites could be attributed to a blocked TCA cycle caused by succinate accumulation or the dysregulation of other enzyme complexes in the TCA cycle. In any case, the results suggest dysregulated mitochondrial function and increased TCA cycle metabolites in the PDLIM2-knockdown lung cancer cells. Succinate and ROS were known to enhance the function of HIF-1α, which is associated with tumor promotion. Mechanistically, accumulated succinate inhibits the enzyme activity of HIF-1α prolyl hydroxylases (PHDs), allowing stabilized HIF-1α to form a complex with HIF-1β for the transcriptional activation of genes that support tumor growth. Similarly, ROS also increase HIF-1α activity for their tumor-promoting effect [15, 16, 24, 25]. These findings are also supported by our observation that H_2_O_2_, a commonly used ROS inducer, increases HIF-1α activity by inhibiting PHD2 expression. Similarly, we treated cells with succinate and dimethyl succinate, which are taken up by cells and directly permeabilize cell membranes, respectively, to elevate intracellular succinate levels, resulting in increased HIF-1α activity by inhibiting PHD2 expression. Furthermore, ROS has been shown to increase the transcription of HIF-1α [55, 56]. Consistent with these findings, the PDLIM2 knockdown cells exhibited elevated mRNA and protein levels of HIF-1α in accompany with the increased levels of succinate and ROS in the cells.

The role of HIF-1α in the tumor-promoting effect of PDLIM2 knockdown had not been reported. The results of our studies, including the discovery of a reverse correlation between PDLIM2 and HIF-1α expressions in lung cancer and the significant reduction of PDLIM2 downregulation-promoted tumor growth with an orally administered HIF-1α inhibitor (PX-478) indicated that increased HIF-1α activity plays a significant role in the growth of lung cancer resulting from PDLIM2 reduction. HIF-1 has been recognized to play a pivotal role in tumor proliferation, angiogenesis, and metastasis, making it an attractive therapeutic target for cancer treatment. Numerous HIF-1 inhibitors have been developed and investigated in clinical trials for the treatment of various cancers, but none of them have been approved for cancer treatment so far. Despite challenges, ongoing efforts continue, with a particular focus on combination therapy and the application of HIF-1 inhibitors to specific types of cancers, such as drug-resistant cancers [82–84].

## Conclusion

In summary, this study discovered a novel mechanism for the pro-tumoral effect of PDLIM2, which involves the accumulation of mitochondrial ROS and oncometabolites leading to the activation of HIF-1α. This funding suggesting PDLIM2 downregulation may serve as a potential marker for therapy with HIF-1 inhibitors, providing new insights into strategies for precise targeted treatment for lung cancer patients with PDLIM2 downregulation.

### Supplementary Information

Supplementary Material 1: Supplementary Fig. S1. Stable PDLIM2 knockdown in lung cancer cells. The efficiency of PDLIM2 knockdown in LLC (A) and A549 (B) lung cancer cell lines was analyzed by qPCR (left panel) and immunoblotting (right panel). Supplementary Fig. S2. Activation of NF-κB suppresses SDH gene expression in lung cancer cells. A, B NF-κB activation was analyzed in control and PDLIM2 knockdown LLC (A) and A549 (B) lung cancer cells by flow cytometry of p65 phosphorylation. C, D LLC (C) and A549 (D) lung cancer cells were pretreated with 5 μM BMS-345541 (BMS) for 20 min prior to TNF-α (50 ng/ml) or IL-1β (50 ng/ml) stimulation for 24 h. The mRNA expression levels of SDH isoforms were measured by real-time PCR. Data represent means ± SEM. * *p* < 0.05, ** *p* < 0.01, *** *p* < 0.001 compared with the control group. Supplementary Fig. S3. Treatment with H_2_O_2_ increases mitochondrial ROS and succinate or dimethyl succinate treatment elevates succinate levels in lung cancer cells. A, B LLC and A549 lung cancer cells were treated with 200 and 400 μM H_2_O_2_ for 2 h. Mitochondrial ROS was analyzed via flow cytometry using the MitoSOX Red probe. C, D LLC and A549 lung cancer cells were treated with 5 mM succinate and 20 mM dimethyl succinate (DMS) for 8 h. Succinate levels in cells were assessed using LC–MS. Data represent means ± SEM. * *p* < 0.05, ** *p* < 0.01, *** *p* < 0.001 compared with control. Supplementary Table S1. Nucleotide sequences of the mouse and human primers used for RT-qPCR in this study.

## Data Availability

The data supporting the fundings of this paper is included in this paper and its supplementary file. The datasets used and /or analyzed in this study are available from the corresponding author on reasonable request.

## Abbreviations

DMEM: Dulbecco's modified Eagle medium
RPMI: Roswell Park Memorial Institute
FACS: Fluorescence-activated cell sorting
FAD: Flavin adenine dinucleotide
FBS: Fetal bovine serum
HIF: Hypoxia-inducible factor
IL: Interleukin
KG: Ketoglutarate
LC-MS: Liquid chromatography-mass spectrometry
LLC: Lewis lung carcinoma
MMP: Matrix metalloproteinase
NAD: Nicotinamide adenine dinucleotide
NF-κB: Nuclear factor kappa-light-chain-enhancer of activated B cells
NOS: Nitric oxide synthase
OCR: Oxygen consumption rate
PDLIM2: PDZ and LIM domain 2
PHD: Prolyl hydroxylase
pVHL: Von Hippel-Lindau tumor suppressor protein
ROS: Reactive oxygen species
SDH: Succinate dehydrogenase
shRNA: Short hairpin RNA
STAT: Signal transducer and activator of transcription
TCA: Tricarboxylic acid
TGF: Transforming growth factor
TNF: Tumor necrosis factor
