# Supplementary material for: Tumor promoting effect of PDLIM2 downregulation involves mitochondrial ROS, oncometabolite accumulations and HIF-1α activation
Source: J Exp Clin Cancer Res. 2024 Jun 17;43:169. doi: 10.1186/s13046-024-03094-9 (PMC11181580; doi:10.1186/s13046-024-03094-9)
Supplement: Supplementary file 1 — Supplementary Material 1: Supplementary Fig. S1. Stable PDLIM2 knockdown in lung cancer cells. The efficiency of PDLIM2 knockdown in LLC (A) and A549 (B) lung cancer cell lines was analyzed by qPCR (left panel) and immunoblotting (right panel). Supplementary Fig. S2. Activation of NF-κB suppresses SDH gene expression in lung cancer cells. A, B NF-κB activation was analyzed in control and PDLIM2 knockdown LLC (A) and A549 (B) lung cancer cells by flow cytometry of p65 phosphorylation. C, D LLC (C) and A549 (D) lung cancer cells were pretreated with 5 μM BMS-345541 (BMS) for 20 min prior to TNF-α (50 ng/ml) or IL-1β (50 ng/ml) stimulation for 24 h. The mRNA expression levels of SDH isoforms were measured by real-time PCR. Data represent means ± SEM. * p < 0.05, ** p < 0.01, *** p < 0.001 compared with the control group. Supplementary Fig. S3. Treatment with H2O2 increases mitochondrial ROS and succinate or dimethyl succinate treatment elevates succinate levels in lung cancer cells. A, B LLC and A549 lung cancer cells were treated with 200 and 400 μM H2O2 for 2 h. Mitochondrial ROS was analyzed via flow cytometry using the MitoSOX Red probe. C, D LLC and A549 lung cancer cells were treated with 5 mM succinate and 20 mM dimethyl succinate (DMS) for 8 h. Succinate levels in cells were assessed using LC–MS. Data represent means ± SEM. * p < 0.05, ** p < 0.01, *** p < 0.001 compared with control. Supplementary Table S1. Nucleotide sequences of the mouse and human primers used for RT-qPCR in this study. [file 13046_2024_3094_MOESM1_ESM.pdf]

Supplementary Material

Tumor promoting effect of PDLIM2 downregulation involves mitochondrial ROS, oncometabolite accumulations and HIF-1α activation

Jing-Xing Yang[1,+], Yu-Chen Chuang [1,+], Jen-Chih Tseng[1], Yi-Ling Liu[1], Chao-Yang Lai [2], Alan Yueh-Luen Lee[3], Chi-Ying F. Huang[4], Yi-Ren Hong[5] and Tsung-Hsien Chuang[1,6,*]

1. Immunology Research Center, National Health Research Institutes, Zhunan, Miaoli 35053, Taiwan.
2. Department of Medical Laboratory Science and Biotechnology, Asia University, Taichung 41354, Taiwan.
3. National Institute of Cancer Research, National Health Research Institutes, Zhunan, Miaoli 35053, Taiwan.
4. Institute of Biopharmaceutical Sciences, College of Pharmaceutical Sciences, National Yang Ming Chiao Tung University, Taipei 11221, Taiwan.
5. Graduate Institute of Medicine, College of Medicine, Kaohsiung Medical University, Kaohsiung 80708, Taiwan.
6. Department of Life Sciences, National Central University, Zhongli District, Taoyuan City 32001, Taiwan.

[+] These authors contributed equally.
* Corresponding author: thchuang@nhri.org.tw

Figure S1

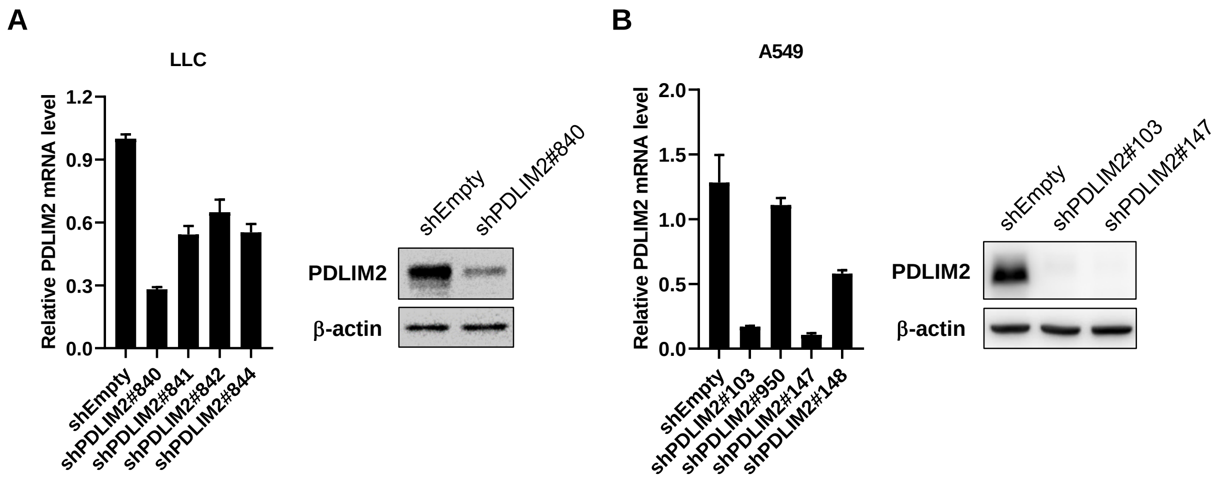

Supplementary Fig. S1 Stable PDLIM2 knockdown in lung cancer cells. The efficiency of PDLIM2 knockdown in LLC (A) and A549 (B) lung cancer cell lines was analyzed by qPCR (left panel) and immunoblotting (right panel).

Figure S2

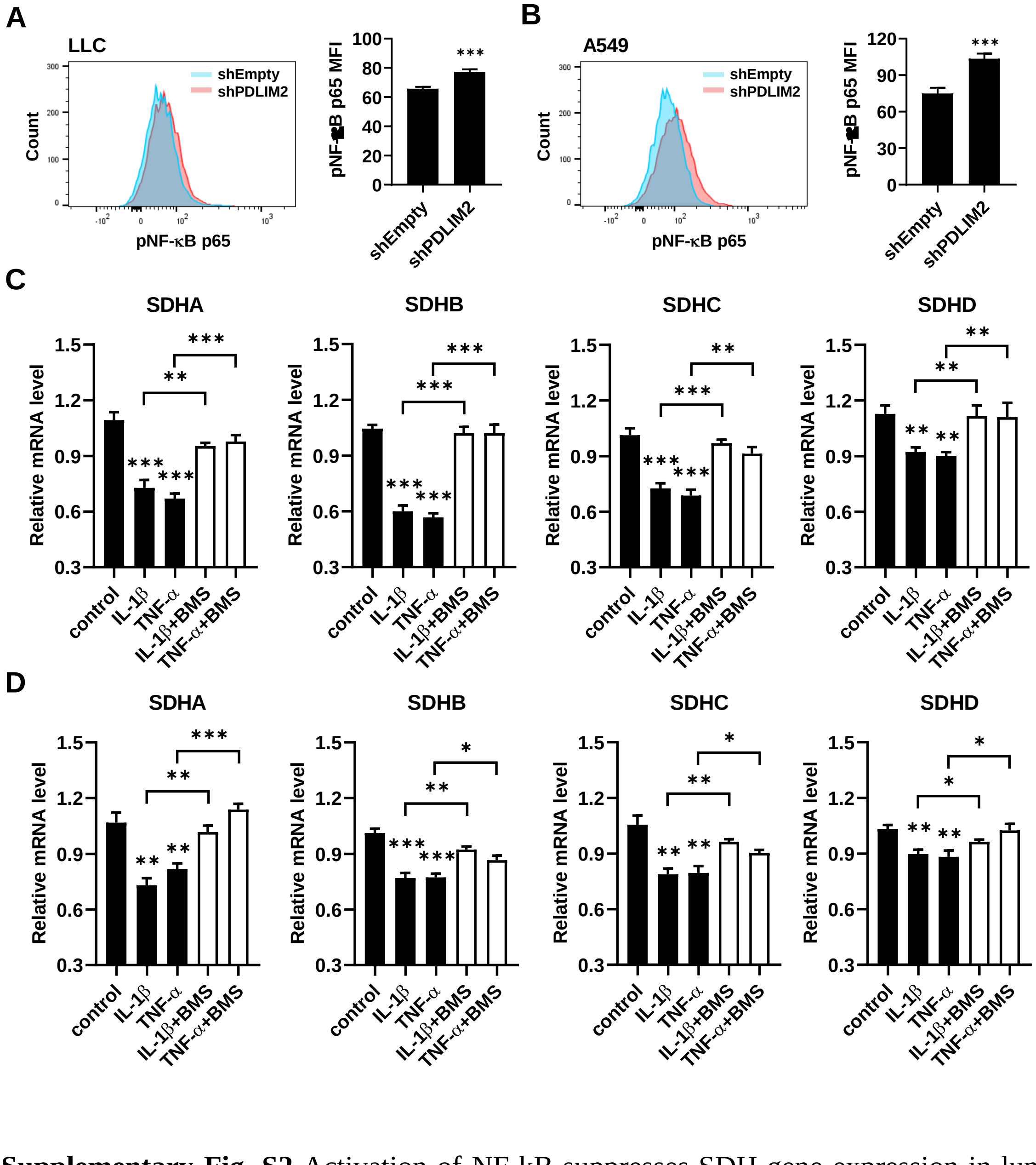

Supplementary Fig. S2 Activation of NF-kB suppresses SDH gene expression in lung cancer cells. **A, B** NF-kB activation was analyzed in control and PDLIM2 knockdown LLC (A) and A549 (B) lung cancer cells by flow cytometry of p65 phosphorylation. **C, D** LLC (C) and A549 (D) lung cancer cells were pretreated with 5 mM BMS-345541 (BMS) for 20 min prior to TNF-α (50 ng/ml) or IL-1β (50 ng/ml) stimulation for 24 h. The mRNA expression levels of SDH isoforms were measured by real-time PCR. Data represent means ± SEM. * $p < 0.05$, ** $p < 0.01$, *** $p < 0.001$ compared with the control group.

Figure S3

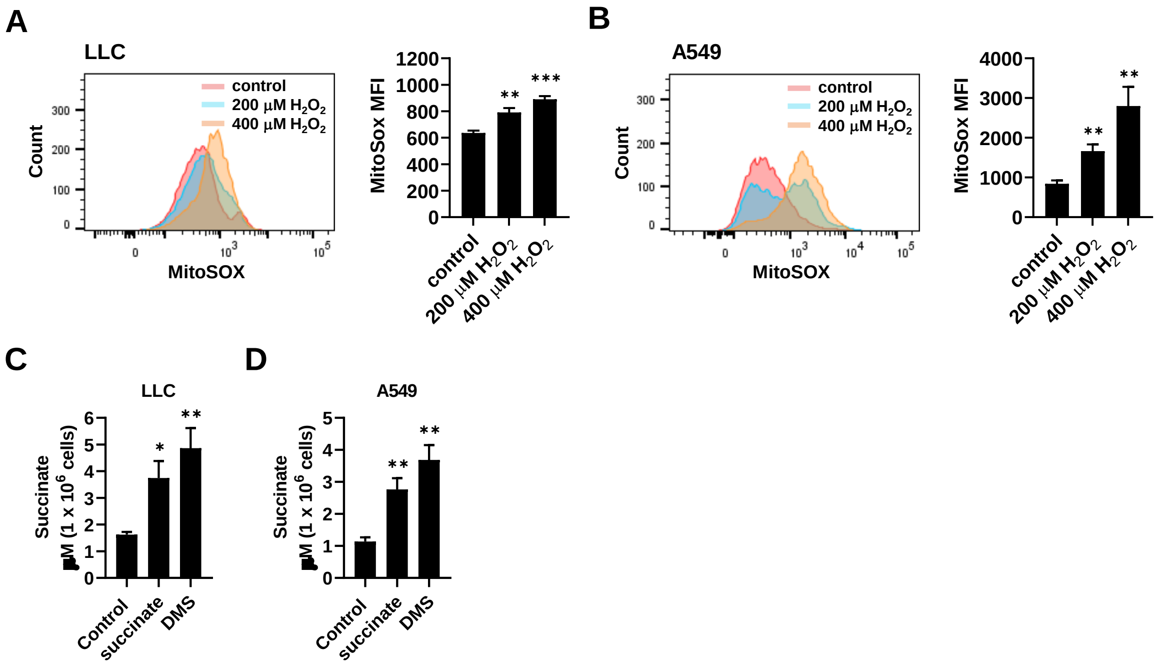

Supplementary Fig. S3 Treatment with H_2O_2 increases mitochondrial ROS, and treatment with succinate or dimethyl succinate elevates succinate levels in lung cancer cells. **A**, **B** LLC and A549 lung cancer cells were treated with 200 and 400 mM H_2O_2 for 2 h. Mitochondrial ROS was analyzed via flow cytometry using the MitoSOX Red probe. **C**, **D** LLC and A549 lung cancer cells were treated with 5 mM succinate and 20 mM dimethyl succinate (DMS) for 8 h. Succinate levels in cells were assessed using LC-MS. Data represent means ± SEM. * $p < 0.05$, ** $p < 0.01$, *** $p < 0.001$ compared with control.

Table S1

Table S1. List of mouse and human qPCR primer sequences used in this study.

Mouse genes

Gene	Forward	Reverse
TNF-α	ACGGCATGGATCTCAAAGAC	GTGGGTGAGGAGCACGTAG
IL-1β	AGAGCTTCAGGCAGGCAGTA	AGGTGCTCATGTCCTCATCC
MMP9	GAAGGCAAACCCTGTGTGTT	AGAGTACTGCTTGCCCAGGA
SDHA	GAGATACGCACCTGTTGCCAAG	GGTAGACGTGATCTTTCTCAGGG
SDHB	TGCGGACCTATGGTGTTGGATG	CCAGAGTATTGCCTCCGTTGATG
SDHC	TGCTCCTTTGGGAACCACAGCT	GCAAACGGACAGTGCCATAGGA
SDHD	GGTTGTCAGTGTTCTGCTCTTGG	GTCGGTAACCACTTGTCCAAGG
PDLIM2	CTTGGAGGCTGAGGAGAGAGGTGGC	CGCCTGGTTCGAGATGTTGACGCTG
HIF-1α	ACAAGTCACCACAGGACAG	AGGGAGAAAATCAAGTCG
β-Actin	CATTGCTGACAGGATGCAGAAGG	TGCTGGAAGGTGGACAGTGAGG

Human genes

Gene	Forward	Reverse
TNF-α	AACCTCCTCTCTGCCATCAA	GGAAGACCCCTCCCAGATAG
IL-1β	GGACAAGCTGAGGAAGATGC	TCGTTATCCCATGTGTCGAA
MMP9	CTCGAACTTTGACAGCGACA	GCCATTCACGTCGTCCTTAT
SDHA	GAGATGTGGTGTCTCGGTCCAT	GCTGTCTCTGAAATGCCAGGCA
SDHB	GCAGTCCATAGAAGAGCGTGAG	TGTCTCCGTTCCACCAGTAGCT
SDHC	GGTTCAAACCGTCCTCTGTCTC	CGACATGCCAAAAAGAGAGACCC
SDHD	GCAGCACATACACTTGTCACCG	GGGAATAGTCCATCGCAGAGCA
PDLIM2	GAGAAGTGCAGTACCAGCATCG	GCATCTTCAGGTTCAGCCCACA
HIF-1α	TATGAGCCAGAAGAACTTTTAGGC	CACCTCTTTTGGCAAGCATCCTG
β-Actin	GGCCAACCGCGAGAAGATGACCCAGA	CGGCCAGAGGCGTACAGGGATAGCA
